# Stimulation of Cysteine-Coated CdSe/ZnS Quantum Dot Luminescence by *meso*-Tetrakis (p-sulfonato-phenyl) Porphyrin

**DOI:** 10.1186/s11671-018-2449-x

**Published:** 2018-02-05

**Authors:** Gustavo G. Parra, Lucimara P. Ferreira, Pablo J. Gonçalves, Svetlana V. Sizova, Vladimir A. Oleinikov, Vladimir N. Morozov, Vladimir A. Kuzmin, Iouri E. Borissevitch

**Affiliations:** 1 Departamento de Física, Faculdade de Filosofia, Ciências e Letras de Ribeirão Preto, Universidade de São Paulo, Ribeirão Preto, SP 14040-901 Brazil; 2 Instituto de Física, Universidade Federal de Goiás, Caixa Postal 131, Goiânia, GO 74001-970 Brazil; 3 Shemyakin-Ovchinnikov Institute of Bioorganic Chemistry RAS, 16/10 Miklukho-Maklaya str, Moscow, Russia 117997; 4 Emanuel Institute of Biophysical Chemistry, RAS-RU, Moscow, Russia; 5 Present Address: MackGraphe, Mackenzie Presbyterian University, São Paulo, SP 01302-907 Brazil

**Keywords:** Luminescence stimulation, Cysteine-coated quantum dot, TPPS_4_ porphyrin, Disaggregation, Electrostatic interaction

## Abstract

Interaction between porphyrins and quantum dots (QD) via energy and/or charge transfer is usually accompanied by reduction of the QD luminescence intensity and lifetime. However, for CdSe/ZnS-Cys QD water solutions, kept at 276 K during 3 months (aged QD), the significant increase in the luminescence intensity at the addition of *meso*-tetrakis (p-sulfonato-phenyl) porphyrin (TPPS_4_) has been observed in this study. Aggregation of QD during the storage provokes reduction in the quantum yield and lifetime of their luminescence. Using steady-state and time-resolved fluorescence techniques, we demonstrated that TPPS_4_ stimulated disaggregation of aged CdSe/ZnS-Cys QD in aqueous solutions, increasing the quantum yield of their luminescence, which finally reached that of the fresh-prepared QD. Disaggregation takes place due to increase in electrostatic repulsion between QD at their binding with negatively charged porphyrin molecules. Binding of just four porphyrin molecules per single QD was sufficient for total QD disaggregation. The analysis of QD luminescence decay curves demonstrated that disaggregation stronger affected the luminescence related with the electron-hole annihilation in the QD shell. The obtained results demonstrate the way to repair aged QD by adding of some molecules or ions to the solutions, stimulating QD disaggregation and restoring their luminescence characteristics, which could be important for QD biomedical applications, such as bioimaging and fluorescence diagnostics. On the other hand, the disaggregation is important for QD applications in biology and medicine since it reduces the size of the particles facilitating their internalization into living cells across the cell membrane.

**Electronic supplementary material:**

The online version of this article (10.1186/s11671-018-2449-x) contains supplementary material, which is available to authorized users.

## Background

Colloidal semiconductor nanocrystals or quantum dots (QD) due to their specific characteristics, intense broad absorption and narrow luminescence spectra with the size-dependent maximum position and high thermal and photostability [1, 2], find applications in various fields of modern technology, such as medical imaging and diagnostics, modern computing nanodevices, fluorescent probes for bioanalytical applications, photoelectrochemical hydrogen generation, etc. ([3–7] and references therein). Functionalization of the QD surface with organic molecules makes it possible to increase their solubility in water, to reduce their toxicity, and to increase their biocompatibility, preparing QD with selective affinity to desirable structures of living organisms [8]. Therefore, QD attract special interest for applications in biology [5] and medicine [6], where they could be successfully applied as fluorescent probes (FP) for fluorescence diagnostics (FD) [9] and photosensitizers (PS) for photochemotherapy (PCT) [10]. Intense absorption in a broad spectral region makes QD an effective antenna for light energy accumulation, and intense narrow luminescence band facilitates the energy transfer to corresponding PS, thus increasing the efficiency of the light energy utilization and consequently increasing the PS efficacy [7, 11]. This makes (QD+PS) pairs promising for application in FD and PCT and stimulates studies in the QD and FS interaction, especially in the transfer of energy and charge between them.

Among others, cysteine-coated (CdSe/ZnS) QD ((CdSe/ZnS)-Cys QD) and *meso*-tetrakis (p-sulfonato-phenyl) porphyrin (TPPS_4_) attract a special interest due to following reasons: a small size of cysteine-coated QD (QD-Cys) which increases its mobility and the probability to penetrate cell membrane, its high chemical stability, low nonspecific adsorption, and high luminescence quantum yield [12, 13]. On the other hand, synthetic TPPS_4_ porphyrin is a promising PS since it is photoactive, water soluble, and non-toxic and has already been tested in clinics in application in photodynamic therapy (PDT) demonstrating hopeful characteristics [14, 15].

Interaction between TPPS_4_ and QD via energy and/or charge transfer has already been documented [16]. Commonly, these processes are accompanied by reduction in the QD luminescence intensity and lifetime. One more process that causes luminescence self-quenching in QD is the self-aggregation via electrostatic interactions or hydrogen bond formation, in many cases, making the aggregation process reversible [17].

In this work, we report for the first time on the stimulation of the QD luminescence via interaction with porphyrin on the example of (CdSe/ZnS)-Cys QD and TPPS_4_ porphyrin.

## Experimental

### Preparation of (CdSe/ZnS)-Cys Quantum Dots

The (CdSe/ZnS)-Cys QD were synthesized in accordance with the method adapted from [18]. The method includes the following: (1) synthesis of colloidal hydrophobic CdSe core nanocrystals and (2) growth of an epitaxial ZnS shell around the CdSe core. To functionalize QD with cysteine, the resultant CdSe/ZnS core–shell QD (~ 3.0 mg) were purified from TOPO via threefold dispersing in chloroform (500 mL) and precipitating with methanol (800 mL). Purified QD were re-dispersed in chloroform (1.0 mL). DL-Cysteine in methanol (30 mg mL^− 1^, 200 mL) was added dropwise to the QD dispersion and mixed vigorously followed by centrifugation (10,000 rpm, 5 min), removing chloroform. After washing with methanol to remove the excess of DL-Cysteine through centrifugation (16,000 rpm, 10 min, 3 times), the QD precipitate was dried under vacuum and re-dispersed in Milli-Q water with 1 M NaOH (20 mL) dropwise addition and filtered with syringe filter Anotop 25 Plus (0.02 μm, Whatman).

### Preparation of porphyrin + (CdSe/ZnS)-Cys QD Samples

The TPPS_4_ porphyrin was obtained from Midcentury Chemicals (USA) and used without additional purification. The experimental solutions were prepared in phosphate buffer (pH 7.3; 7.5 mM), using Milli-Q quality water. For luminescence measurements in (CdSe/ZnS)-Cys QD kept at 276 K for 3 months (aged QD), aliquots of a concentrated TPPS_4_ stock solution ([TPPS_4_]_stock_ = 140 μM) were added to the (CdSe/ZnS)-Cys QD initial solution, avoiding dilution effects. For the aged QD dilution experiment, aliquots of the initial solution were replaced by the same amount of phosphate buffer. All the experiments were performed at room temperature (297 K).

The concentration of TPPS_4_ was controlled spectrophotometrically using ε_515nm_ = 1.3 × 10^4^ M^− 1^ cm^− 1^ [19]. The concentration of the aged (CdSe/ZnS)-Cys quantum dot was calculated using the first excitonic absorption peak at 520 nm using *ε* = 5857(*D*)^2.65^ according to Yu’s empirical calculation [20], where *D*(nm) is the diameter of the given nanocrystal. The *D* value was determined from the empirical fitting function of the curve as presented in [20]. For CdSe nanocrystals, this function is:

1 $$ D=\left(1.6122\times {10}^{-9}\right){\lambda}^4-\left(2.6575\times {10}^{-6}\right){\lambda}^3+\left(1.6242\times {10}^{-3}\right){\lambda}^2-(0.4277)\lambda +(41.57) $$

In our case, *λ* = 520 nm, *D* = 2.6 nm, and *ε* = 7.4 × 10^4^ M^− 1^ cm^− 1^.

### Instruments

The absorption spectra were monitored with a Beckman Coulter DU640 spectrophotometer. The steady-state luminescence measurements were made on a Hitachi F-7000 spectrofluorimeter at *λ*_ex_ = 480 nm and *λ*_em_ = 558 nm. The aged QD luminescence quantum yield (QY) was determined via relative method [21] with a single point measurement, *λ*_ex_ = 480 nm and *λ*_em_ = 558 nm, using 1-palmitoyl,2-(12-[N-(7-nitrobenz-2-oxa-1,3-diazol-4-yl)amino]dodecanoyl)-sn-glycero-3-phosphocholine (C12-NBD-PC) as a standard (QY = 0.34 in ethanol) [22, 23] according to the equation:

2 $$ {\Phi}_{fl}={\Phi}_{fl0}\frac{n^2{I}_{fl}}{n_0^2{I}_{fl0}}\frac{A_0}{A} $$

where *I*_fl_ and *I*_fl0_ are the integral fluorescence intensities of QD and C12-NBD-PC, *A* and *A*_0_ are their absorbances at *λ*_ex_ = 480 nm, and *n* and *n*_*0*_ are refractive indexes of the used solvents, respectively.

Time-resolved experiments were carried out by using an apparatus based on the time-correlated single-photon counting method. The excitation source was a Tsunami 3950 Spectra Physics titanium-sapphire laser, pumped by a Millenia X Spectra Physics solid state laser. The frequency of the laser pulse repetition was 8.0 MHz using the 3980 Spectra Physics pulse picker. The laser was tuned so that the second harmonic generator BBO crystal (GWN-23PL Spectra Physics) gave the 480 nm excitation pulses that were directed to an Edinburgh FL900 spectrometer. The spectrometer was in L-format configuration, the emission wavelength was selected by a monochromator, and the emitted photons were detected by a refrigerated Hamamatsu R3809U microchannel plate photomultiplier. The full width at half maximum (FWHM) of the instrument response function was typically 100 ps, and time resolution was 12 ps per channel. The software provided by Edinburgh Instruments and the commercial “OriginPro9” software were used to fit the experimental luminescence decay curves.

The quality of the fit was evaluated by the analysis of the statistical parameter reduced-*χ*^2^ and by inspection of the residuals distribution.

Dynamic light scattering was measured with NanoBrook 90Plus Zeta Particle Size Analyzer with excitation at 640 nm using a 40 mW HeNe laser (Brookhaven Instruments Corporation).

## Results and Discussion

**Fig. 1:**
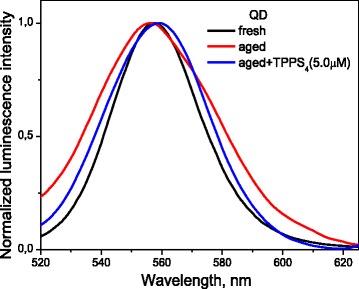
Normalized luminescence spectra of (CdSe/ZnS)-Cys 558 quantum dots in phosphate buffer (7.5 mM) at pH 7.3: fresh prepared (black line, *λ*_max_ = 558 nm), after 3 months in the refrigerator at 276 K (aged QD) without TPPS_4_ (red line, *λ*_max_ = 556 nm), and at addition of [TPPS_4_] = 5.0 μM to aged QD (blue line, *λ*_max_ = 559 nm), *λ*_ex_ = 480 nm

Freshly prepared (CdSe/ZnS)-Cys QD possess the maximum of the luminescence spectrum at 558 nm (Fig. 1, black line), as previously reported by Liu et al. [13], and quantum yield (QY) 0.75 [2, 24, 25]. The addition of TPPS_4_ to the fresh solution induces no changes, both in the QD luminescence intensity and the luminescence spectrum profile.

For (CdSe/ZnS)-Cys QD dissolved in water and kept in the refrigerator at 276 K for 3 months (aged QD), the position of the maximum of the luminescence spectrum, measured in phosphate buffer (7.5 mM) at pH 7.3, was blue-shifted for 2 nm (*λ*_max_ = 556 nm), as compared with that of fresh QD. The emission band appeared widened and slightly asymmetric (Fig. 1, red line). The quantum yield of the aged QD luminescence, determined by the method described above, was 0.23 ± 0.03.

**Fig. 2:**
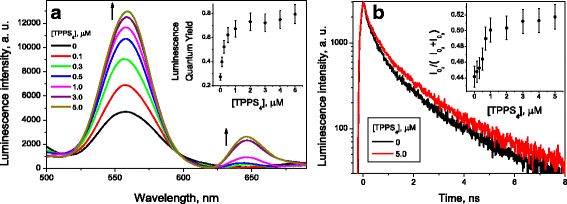
**a** Luminescence spectra and quantum yield (inset) of aged (CdSe/ZnS)-Cys 558 QD ([QD] = 570 nM, black curve) solutions as a function of the TPPS_4_ porphyrin concentration. **b** Decay kinetics of QD luminescence and the ${I}_{0_3}/\left({I}_{0_2}+{I}_{0_3}\right)$ ratio (inset, see Eq. (3)) as a function of the TPPS_4_ porphyrin concentration

The addition of TPPS_4_ to the aged QD solution induced significant increase in the luminescence intensity (Fig. 2a), QY value reaching 0.75 ± 0.08 (Fig. 2a, inset), the value closed to that for fresh QD [2, 24, 25].

Moreover, in the presence of TPPS_4_, the symmetrization of the luminescence band of the aged QD and the reduction of its bandwidth were observed, accompanied by maximum red shift up to *λ*_max_ = 559 nm, close to the maximum of the fresh QD spectrum (Fig. 1, blue line.).

The luminescence decay curves obtained at 480 nm excitation for solutions of both fresh and aged QD were successively fitted as a sum of three exponentials:

3 $$ I={I}_{0_1}{e}^{-t/{\tau}_1}+{I}_{0_2}{e}^{-t/{\tau}_2}+{I}_{0_3}{e}^{-t/{\tau}_3} $$

where ${I}_{0_i}$ and *τ*_*i*_ are pre-exponential factor (amplitude) and lifetime of the *i*-th decay component, respectively.

The component lifetimes for both fresh and aged QD are independent from the porphyrin presence (Table 1). The luminescence lifetimes of fresh QD solutions are typical for (CdSe/ZnS)-Cys QD [26, 27]. For aged QD, the component lifetimes are much shorter (Table 1).

**Table 1:** Lifetimes of the (CdSe/ZnS)-Cys QD luminescence decay curve components in phosphate buffer (7.5 mM) at pH 7.3: fresh QD and those after 3 months in the refrigerator at 276 K (aged QD) in the absence and presence of TPPS_4_, *λ*_ex_ = 480 nm, *λ*_em_ = 558 nm

Sample	τ_1_, ns	τ_2_, ns	τ_3_, ns
Fresh QD	0.7 ± 0.1	3.8 ± 0.4	19 ± 1
Fresh QD + 8.6μМ [TPPS_4_]	0.7 ± 0.1	4.1 ± 0.5	20 ± 1
Aged QD	0.1 ± 0.1	1.0 ± 0.2	5.2 ± 0.5
Aged QD + 5.0μМ [TPPS_4_]	0.1 ± 0.1	0.9 ± 0.2	5.2 ± 0.5

The values of *τ*_1_ in all cases, fresh and aged QD in the presence and absence of porphyrin, are close to time resolution of the single-photon counting equipment (≈ 100 ps) used in this study. Therefore, it should be associated with the scattered light of the exciting pulse.

It is well established [28–30] that the short-lived (*τ*_2_) and long-lived (*τ*_3_) components are associated with the luminescence resulted from the electron-hole annihilation in the QD core (*τ*_2_) and shell (*τ*_3_), respectively. Total intensity of these two components characterizes the whole annihilation process in the QD. In this case, the relative intensity (amplitude) of the *τ*_3_ component should demonstrate the contribution of the electron-hole annihilation in the QD shell. The relative contribution *I*_3_ of 3rd component to the decay curve was calculated as:

4 $$ {I}_3=\frac{I_{0_3}}{I_{0_2}+{I}_{0_3}} $$

The addition of TPPS_4_ to fresh QD solutions does not change significantly the relative content of the components (data not shown), while for aged QD solutions, the relative content of *τ*_3_ component *I*_3_ increases with the TPPS_4_ concentration (Fig. 2b, inset). The dependence of QY for aged quantum dot luminescence on the TPPS_4_ concentration is similar to that for *I*_3_ (Fig. 2a, b, insets), both reaching the maximum values approximately at 2.0 μM TPPS_4_. This means that TPPS_4_ interacting with aged QD affects stronger the luminescence of QD shell than that of its core. However, TPPS_4_ in fresh QD solutions demonstrates no effect upon the QD luminescence. Therefore, we conclude that the TPPS_4_ effect observed for the aged QD solution cannot be explained by the porphyrin binding to the QD surface.

On the other hand, the observed increase of the aged QD luminescence intensity at interaction with TPPS_4_ cannot be explained via reverse energy transfer from TPPS_4_ to QD, since the TPPS_4_ fluorescence spectrum is localized in the range *λ* > 600 nm where QD absorption is weak (Additional file 1: Figure S3). Therefore, the energy transfer via Förster resonance energy transfer (FRET) mechanism is low probable. Moreover, the QD luminescence was excited at 460 or 480 nm, where TPPS_4_ optical absorption is negligible. In addition, the absorption spectra of TPPS_4_ remained unchanged in the mixed solutions, demonstrating the absence of charge transfer between QD and TPPS_4_ (Additional file 1: Figure S4b, c).

The ability of quantum dots to aggregate via formation of non-covalent NH···H hydrogen bonds between the QD surface groups was already documented [13, 17]. Aggregation reduces the QD luminescence, quenching most effectively the component attributed to the QD shell [13, 17]. The reduction of the QD luminescence intensity and lifetime was observed for CdSe-QD in solid films due to formation of 3D aggregates [31]. The authors proposed a model, where this reduction is associated with energy transfer between individual QD in the aggregate [32].

On the bases of this evidence, we believe that while in the refrigerator, QD do aggregate, which reduces the luminescence intensity and lifetimes. Therefore, we associate the observed increase in the QD luminescence intensity and lifetimes in the presence of TPPS_4_ with QD disaggregation, stimulated by TPPS_4_ at its binding with the aggregate. The similar effect was observed for the emission of aggregated QD at their interaction with fluorine ions [17].

The observed changes in the luminescence band profile for aged QD (Fig. 1) can be explained by QD aggregation, as well, its asymmetry being associated with existence of different types of aggregates. Interaction with TPPS_4_ reduces aggregation and makes the luminescence band profile similar to that for non-aggregated QD, observed in fresh solutions.

**Fig. 3:**
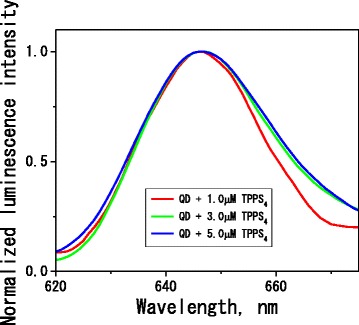
Normalized luminescence emission spectra of TPPS_4_ in phosphate buffer (7.5 mM, pH 7.3) for various TPPS_4_ concentrations in the presence of aged (CdSe/ZnS)-Cys 558 quantum dot (570 nM), *λ*_ex_ = 460 nm

At neutral pH, the QD-Cys surface possesses negative net charge due to deprotonation of terminal amino groups on its surface [17, 33, 34]. At this pH, TPPS_4_ has net charge (4-) due to four negatively charged sulfonate phenyl groups in its structure ([35, 36] and references therein). Therefore, interaction between QD cysteine groups and TPPS_4_ molecules is low probable because of electrostatic repulsion. However, high affinity of the porphyrin π-conjugated system for metal surfaces is well documented [37]. This affinity should be responsible for TPPS_4_ binding on the quantum dots surface, despite of the electrostatic repulsion between the QD and porphyrin side groups. The interaction between QD surface and π-conjugated system of the bound porphyrin could explain weak broadening of the porphyrin fluorescence spectrum (Figs. 1, 3, and Additional file 1: Figure S3a, inset) and observed changes in the fluorescence excitation spectrum (Additional file 1: Figure S5b, inset) [38].

**Scheme 1:**
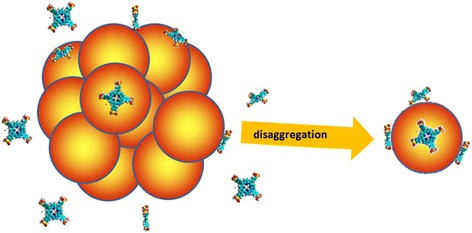
The scheme of the interaction between aged (CdSe/ZnS)-Cys 558 QD and TPPS_4_ porphyrin at neutral pH. The porphyrin molecules adsorb on the QD surface due to the high affinity of the porphyrin π-conjugated system for metal surfaces increasing the net negative charge on the QD surface, thus increasing electrostatic repulsion between particles and inducing their disaggregation

Binding of some porphyrin molecules on the QD surface increases the QD surface negative charge, thus increasing electrostatic repulsion between particles and inducing their disaggregation (Scheme 1) [39].

The QD surface area *A*_QD_ ≈ 145 nm^2^ is sufficient to adsorb several TPPS_4_ molecules (*A*_TPPS4_ ≈ 1.8 nm^2^ per unit) [40], as it was observed for porphyrins interacting with magnetic and gold nanoparticles [41, 42].

To cover the whole area of QD by porphyrins, 80 porphyrin molecules per individual QD are necessary. However, the saturation of the luminescence QY and *I*_3_ values in 570 nM QD solution was observed at approximately [TPPS_4_] = 2.0 μM (Fig. 2), which demonstrates that the binding of four porphyrin molecules per QD is sufficient for QD disaggregation. This could be explained by larger charge density on the porphyrin molecule as compared with that of QD (Additional file 1: Figure S6) which produces stronger electrostatic repulsion between QD with bound porphyrins. Indeed, Zeta-potential for the aged QD (ζ_QD_) is − 36.1 mV and that for TPPS_4_ molecule (ζ_TPPS4_) is − 37.6 mV. Average charge density, calculated as σ = ζ /A_QD_, for an individual aged QD is

σ_QD_ = − 36.1 mV/145 nm^2^ = − 0.25 mV/nm^2^.

At the same time, for an individual aged QD bound with four TPPS_4_ molecules, the average charge density (σ_QD+TPPS4_) is

σ_QD+TPPS4_ = − (36.1 + 37.6 × 4) mV/145 nm^2^ = − 1.29 mV/nm^2^.

Thus, the binding of four TPPS_4_ molecules with an individual aged QD increases its σ more than 5 times, increasing the force of electrostatic repulsion more than 25 times and inducing the aged QD disaggregation.

**Fig. 4:**
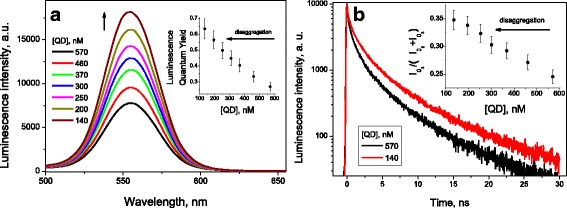
**a** Luminescence spectra and quantum yield (inset) of aged (CdSe/ZnS)-Cys 558 QD solutions in function of its concentration. **b** Decay kinetics of the QD luminescence and the *I*_3_ value (inset, see Eq. (3)) in function of its concentration

In accordance with the QD aggregation hypothesis, a similar effect to that induced by TPPS_4_ addition should be observed at dilution of aged QD solutions. Really, we have observed the increase in the QY of QD luminescence at dilution of its buffer solution (Fig. 4a, inset), which demonstrates that the self-quenching of QD luminescence in aged QD-Cys solutions depends on QD concentration [17]. Simultaneously, the *I*_3_ value in the QD luminescence kinetics increases with dilution, as well (Fig. 4b, inset).

Moreover, the dynamic light scattering experiments show that D_hd_ of scattering particles in the QD solutions after aging was (330 ± 170) nm, which is much larger than that of fresh QD. Dilution reduces D_hd_ down to (25 ± 6) nm, thus demonstrating directly the QD disaggregation (Additional file 1: Table S1).

There exists one more interesting aspect of the problem: can addition of TPPS_4_ to the solution of fresh QD prevent their aggregation during storage at low temperature, thus stabilizing their luminescence characteristics? However, clarification of this problem needs an independent and detailed study using various experimental methods and varying experimental conditions, such as reagent concentrations, temperature, duration of the solution storage (several months), etc. We plan to realize this profound study in the nearest future.

## Conclusions

Basing on the obtained data, we can assert that the long storage of CdSe/ZnS-Cys QD in aqueous solutions even at low temperatures induces their aggregation, which reduces the luminescence quantum yield and lifetimes. The addition of TPPS_4_ porphyrin stimulates disaggregation of aged CdSe/ZnS-Cys QD which is pronounced via increase of the QD luminescence quantum yield and the contribution of electron-hole annihilation in the QD shell in the total QD luminescence. The disaggregation, stimulated by porphyrin, takes place due to the increase of electrostatic repulsion between aggregated QD at their binding with negatively charged porphyrin molecules. Disaggregation has been observed at the dilution of QD solution, as well.

The obtained results demonstrate the way to repair the aged QD by adding some molecules or ions to solutions, stimulating QD disaggregation and restoring their luminescence characteristics, which could be important for QD biomedical applications, such as bioimaging and fluorescence diagnostics. On the other hand, disaggregation is important for QD applications in biology and medicine since it reduces the size of the particles facilitating their internalization into living cells across the cell membrane.

## Additional file

Additional file 1: The experimental solutions were prepared in phosphate buffer (pH7.3;7.5mM), using Milli-Q quality water. **Figure S1**. Dynamic light scattering diagram of fresh prepared (CdSe/ZnS)-Cys 558 quantum dots (QD). **Figure S2**. Luminescence decay curve of freshly prepared (CdSe/ZnS)-Cys 558 QD solution; λ_ex_ =480nm and λ_em_ =558nm. **Figure S3**. **a** Normalized optical absorption spectrum of non-protonated TPPS_4_ . **Inset**: Normalized optical absorption spectra of the TPPS_4_ Q-bands (black line) and “aged” QD (red line), and the fluorescence emission spectrum of non-protonated TPPS_4_ with maximum at 644nm (blue line), λ_ex_ =515nm. **b** TPPS_4_ fluorescence decay kinetics at 650nm, λ_ex_ =515nm. **Figure S4**. **a** Optical absorption spectra of the aged (CdSe/ZnS)-Cys 558 QD and TPPS_4_ mixture at different TPPS_4_ concentrations. **Inset**: Details of the absorption spectra in the region of the porphyrin Q-bands. **b** Optical absorption spectra just for TPPS_4_ in the mixture TPPS_4_ +QD. The final spectrum of each sample was obtained subtracting the initial QD absorption spectrum (no TPPS_4_ adding). **c** Details of the absorption spectra in the region of the porphyrin Qbands, showing that TPPS_4_ absorption spectrum does not change in the presence of aged (CdSe/ZnS)-Cys 558 QD. The curves for 0.1 and 0.3μM of TPPS_4_ are not shown due to the lower signal-to-noise ratio of Qbands. No significant spectral shift was observed either Soret or Q-bands. **Figure S5**. **a** Normalized fluorescence excitation spectra of TPPS_4_ in Milli-Q quality water as a function of TPPS_4_ concentrations, λ_em_ =646nm. **b** Luminescence excitation spectra of TPPS_4_ and aged (CdSe/ZnS)-Cys 558 QD mixtures as a function of TPPS_4_ concentrations; λ_em_ =646nm; [QD]=570nM. **Figure S6**. **a** Zeta-potential measured on Malvern ZETASIZER 3000HSA (λ_ex_ =633nm, 10mW HeNe laser) **a** aged QD (ξ_aged-QD_) and **b** TPPS_4_ porphyrin (ξ_TPPS4_). **Table S1**. Variation of aged (CdSe/ZnS)-Cys 558 QD hydrodynamic diameter (D_hd_) as a function of its concentration measured on NanoBrook 90Plus Zeta Particle Size Analyzer (λ_ex_ =640nm, 40mW HeNe laser). (PDF 524 kb)

## Abbreviations

C12-NBD-PC: 1-Palmitoyl,2-(12-[N-(7-nitrobenz-2-oxa-1,3-diazol-4-yl)amino]dodecanoyl)-sn-glycero-3-phosphocholine
FD: Fluorescence diagnostics
FP: Fluorescent probes
FWHM: Full width at half maximum
PCT: Photochemotherapy
PDT: Photodynamic therapy
PS: Photosensitizers
QD: Quantum dots
QD-Cys: Cysteine-coated QD
QY: Quantum yields
TOPO: Trioctylphosphine oxide
TPPS_4__4_: *meso*-tetrakis (p-sulfonato-phenyl) porphyrin
