# Supplementary material for: Stimulation of Cysteine-Coated CdSe/ZnS Quantum Dot Luminescence by meso-Tetrakis (p-sulfonato-phenyl) Porphyrin
Source: Nanoscale Res Lett. 2018 Feb 5;13:40. doi: 10.1186/s11671-018-2449-x (PMC5799094; doi:10.1186/s11671-018-2449-x)
Supplement: Additional file 1: — The experimental solutions were prepared in phosphate buffer (pH7.3;7.5mM), using Milli-Q quality water. Figure S1. Dynamic light scattering diagram of fresh prepared (CdSe/ZnS)-Cys 558 quantum dots (QD). Figure S2. Luminescence decay curve of freshly prepared (CdSe/ZnS)-Cys 558 QD solution; λex =480nm and λem =558nm. Figure S3. a Normalized optical absorption spectrum of non-protonated TPPS4 . Inset: Normalized optical absorption spectra of the TPPS4 Q-bands (black line) and “aged” QD (red line), and the fluorescence emission spectrum of non-protonated TPPS4 with maximum at 644nm (blue line), λex =515nm. b TPPS4 fluorescence decay kinetics at 650nm, λex =515nm. Figure S4. a Optical absorption spectra of the aged (CdSe/ZnS)-Cys 558 QD and TPPS4 mixture at different TPPS4 concentrations. Inset: Details of the absorption spectra in the region of the porphyrin Q-bands. b Optical absorption spectra just for TPPS4 in the mixture TPPS4 +QD. The final spectrum of each sample was obtained subtracting the initial QD absorption spectrum (no TPPS4 adding). c Details of the absorption spectra in the region of the porphyrin Qbands, showing that TPPS4 absorption spectrum does not change in the presence of aged (CdSe/ZnS)-Cys 558 QD. The curves for 0.1 and 0.3μM of TPPS4 are not shown due to the lower signal-to-noise ratio of Qbands. No significant spectral shift was observed either Soret or Q-bands. Figure S5. a Normalized fluorescence excitation spectra of TPPS4 in Milli-Q quality water as a function of TPPS4 concentrations, λem =646nm. b Luminescence excitation spectra of TPPS4 and aged (CdSe/ZnS)-Cys 558 QD mixtures as a function of TPPS4 concentrations; λem =646nm; [QD]=570nM. Figure S6. a Zeta-potential measured on Malvern ZETASIZER 3000HSA (λex =633nm, 10mW HeNe laser) a aged QD (ξaged-QD) and b TPPS4 porphyrin (ξTPPS4). Table S1. Variation of aged (CdSe/ZnS)-Cys 558 QD hydrodynamic diameter (Dhd) as a function of its concentration measured on NanoBrook 90Plus Zeta Particle [file 11671_2018_2449_MOESM1_ESM.pdf]

Supplementary data

Stimulation of cysteine-coated CdSe/ZnS quantum dot luminescence by *meso*-tetrakis (p-sulfonato-phenyl) porphyrin

Gustavo G. Parra[1,5*], Lucimara P. Ferreira[1], Pablo J. Gonçalves[2], Svetlana V.Sizova[3], Vladimir A. Oleinikov[3], Vladimir N. Morozov[4], Vladimir A. Kuzmin[4], and Iouri E. Borissevitch[1,2]

[1] *Departamento de Física, Faculdade de Filosofia, Ciências e Letras de Ribeirão Preto, Universidade de São Paulo, Ribeirão Preto, SP, Brazil 14040-901*

[2] *Instituto de Física, Universidade Federal de Goiás, Caixa Postal 131, 74001-970 Goiânia, GO, Brazil*

[3] *Shemyakin-Ovchinnikov Institute of Bioorganic Chemistry RAS, 117997 Moscow, 16/10 Miklukho-Maklaya str., Russia*

[4] *Emanuel Institute of Biophysical Chemistry, RAS-RU, Moscow, Russia.*

[5] *Present Address: MackGraphe, Mackenzie Presbyterian University, São Paulo, SP 01302-907, Brazil*

* *Corresponding authors:* Gustavo G. Parra (gugparra@gmail.com)

The experimental solutions were prepared in phosphate buffer (pH7.3;7.5mM), using Milli-Q quality water.

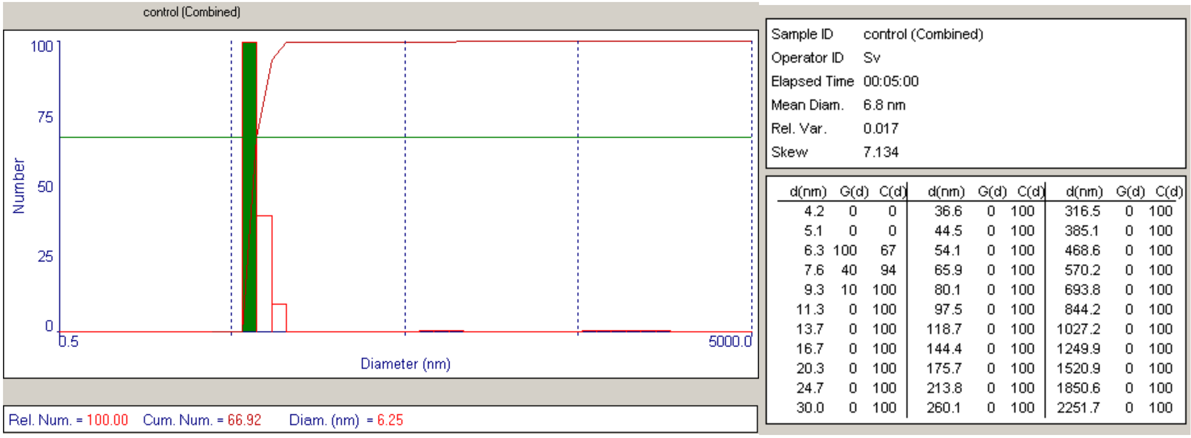

Figure S1. Dynamic light scattering diagram of fresh prepared (CdSe/ZnS)-Cys 558 quantum dots (QD).

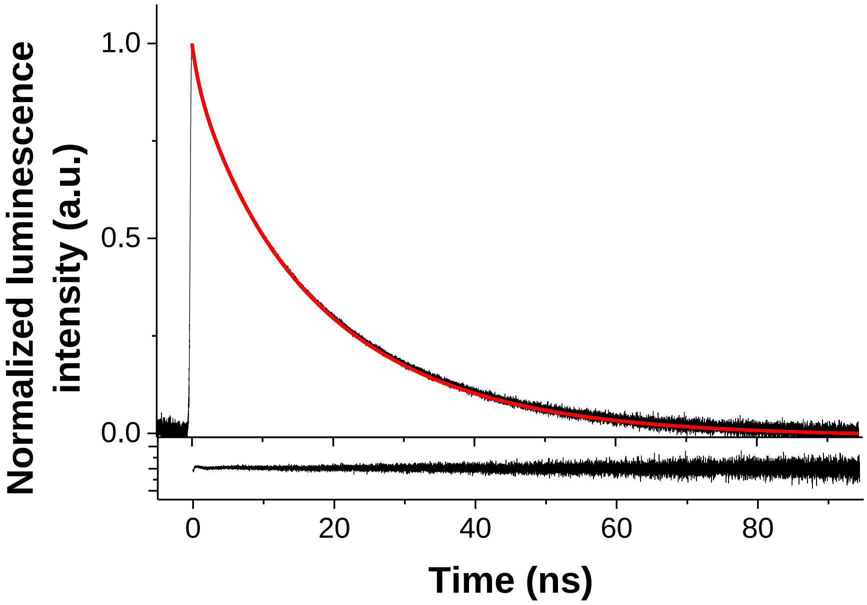

Figure S2. Luminescence decay curve of freshly prepared (CdSe/ZnS)-Cys 558 QD solution; λ_{ex}=480nm and λ_{em}=558nm.

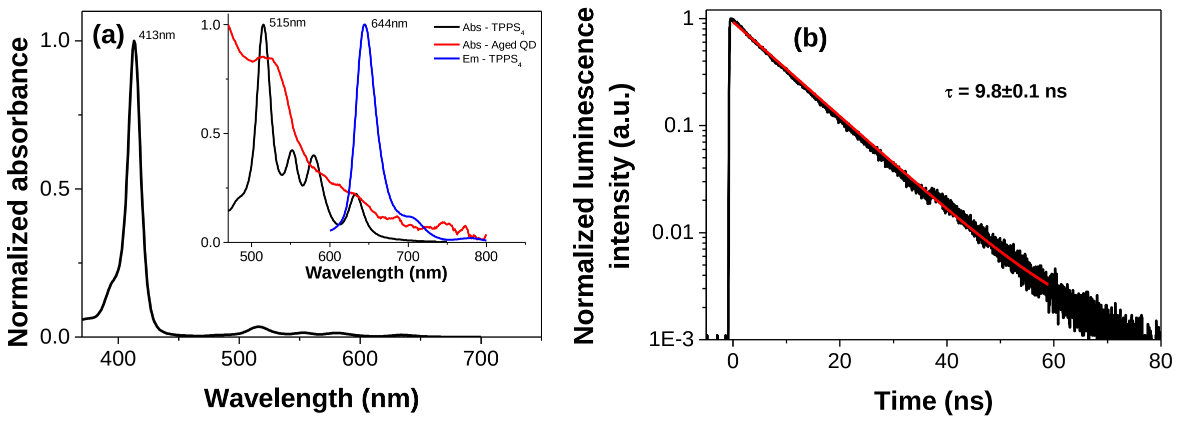

Figure S3. a Normalized optical absorption spectrum of non-protonated $TPPS_4$. **Inset:** Normalized optical absorption spectra of the $TPPS_4$ Q-bands (black line) and "aged" QD (red line), and the fluorescence emission spectrum of non-protonated $TPPS_4$ with maximum at 644nm (blue line), λ_{ex}=515nm. **b** $TPPS_4$ fluorescence decay kinetics at 650nm, λ_{ex}=515nm.

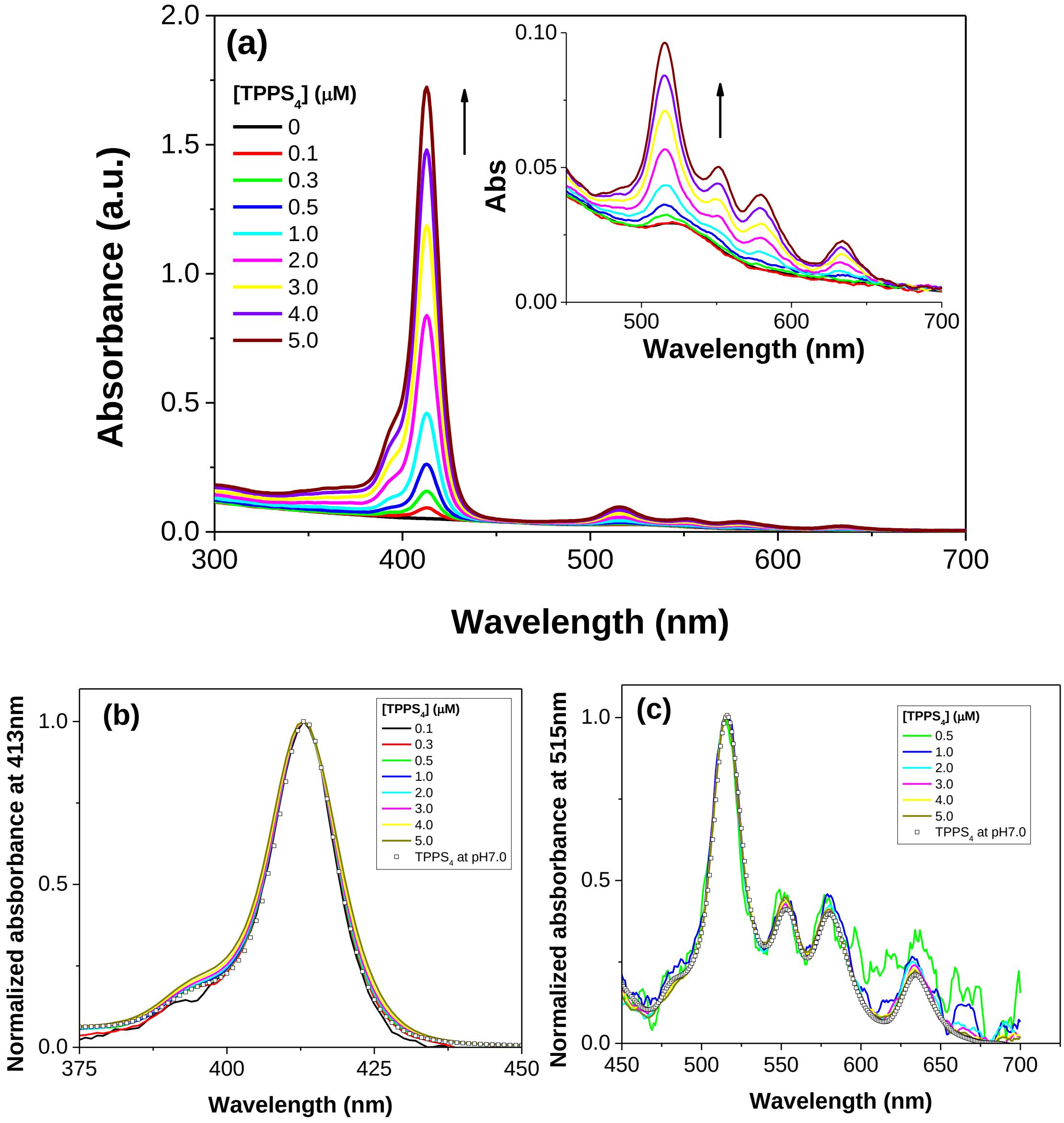

Figure S4. a Optical absorption spectra of the aged (CdSe/ZnS)-Cys 558 QD and $TPPS_4$ mixture at different $TPPS_4$ concentrations. **Inset:** Details of the absorption spectra in the region of the porphyrin Q-bands. **b** Optical absorption spectra just for $TPPS_4$ in the mixture $TPPS_4$+QD. The final spectrum of each sample was obtained subtracting the initial QD absorption spectrum (no $TPPS_4$ adding).

c Details of the absorption spectra in the region of the porphyrin Q-bands, showing that $TPPS_4$ absorption spectrum does not change in the presence of aged (CdSe/ZnS)-Cys 558 QD. The curves for 0.1 and 0.3μM of $TPPS_4$ are not shown due to the lower signal-to-noise ratio of Q-bands. No significant spectral shift was observed either Soret or Q-bands.

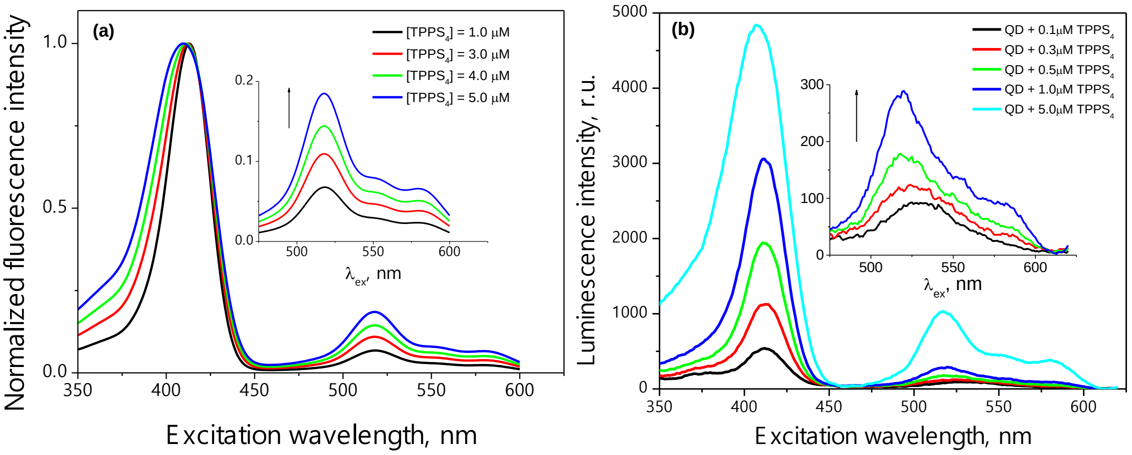

Figure S5. a Normalized fluorescence excitation spectra of $TPPS_4$ in Milli-Q quality water as a function of $TPPS_4$ concentrations, λ_{em}=646nm. **b** Luminescence excitation spectra of $TPPS_4$ and aged (CdSe/ZnS)-Cys 558 QD mixtures as a function of $TPPS_4$ concentrations; λ_{em}=646nm; [QD]=570nM.

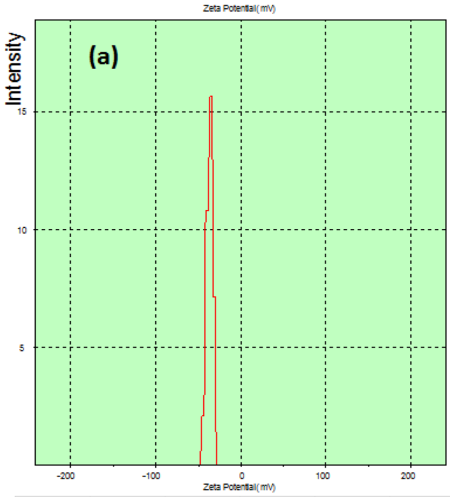

Run	Pos.	KCps	Mob.	Zeta	Width
1	50.0	945.7	-2.683	-33.8	12.9
2	50.0	917.0	-2.862	-36.1	12.4
3	50.0	945.6	-2.831	-35.7	1.6
4	50.0	963.9	-2.911	-36.7	1.6
5	50.0	939.6	-2.928	-36.9	1.6
6	50.0	931.9	-2.901	-36.6	1.6
7	50.0	940.9	-2.851	-36.0	1.6
8	50.0	928.8	-2.842	-35.9	1.6
9	50.0	911.2	-2.866	-36.2	1.6
10	50.0	910.2	-2.914	-36.8	1.6
Average		933.5	-2.859	-36.1	3.8
+/-		17.2	0.070	0.9	4.7

(b)

Results

			Mean (mV)	Area (%)	St Dev (mV)
**Zeta Potential (mV):**	-37,6	**Peak 1:**	-38,7	95,8	5,27
**Zeta Deviation (mV):**	6,33	**Peak 2:**	-13,7	4,2	2,14
**Conductivity (mS/cm):**	0,0415	**Peak 3:**	0,00	0,0	0,00
**Result quality**	Good				

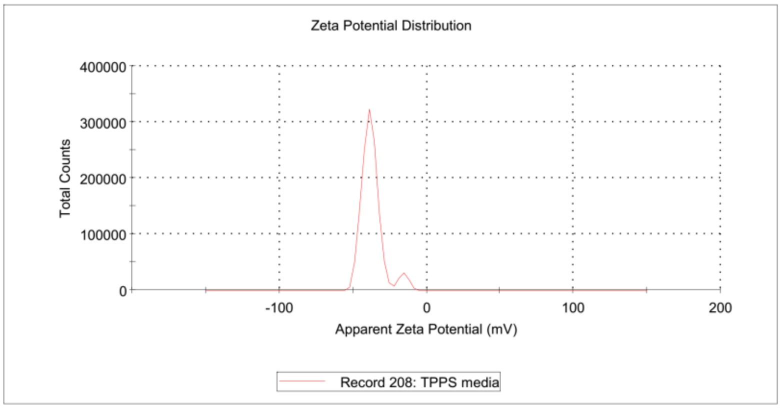

Figure S6. Zeta-potential measured on Malvern ZETASIZER 3000HSA (λ_{ex}=633nm, 10mW HeNe laser) **a** aged QD ($\xi_{aged\text{-}QD}$) and **b** $TPPS_4$ porphyrin (ξ_{TPPS4}).

Table S1. Variation of aged (CdSe/ZnS)-Cys 558 QD hydrodynamic diameter (D_{hd}) as a function of its concentration measured on NanoBrook 90Plus Zeta Particle Size Analyzer (λ_{ex}=640nm, 40mW HeNe laser).

[aged QD] (nM)	D_{hd} (nm)
370	330±170
300	170±70
250	150±70
200	25±6
